# A novel *FGFR2* (S137W) mutation resulting in Apert syndrome: A case report

**DOI:** 10.1097/MD.0000000000022340

**Published:** 2020-09-25

**Authors:** Qingyang Shi, Rulin Dai, Ruixue Wang, Jili Jing, Xiaowei Yu, Ruizhi Liu, Yanhong Liu

**Affiliations:** Center of Reproductive Medicine and Center of Prenatal Diagnosis, the First Hospital, Jilin University, Changchun, Jilin, China.

**Keywords:** Apert syndrome, autosomal dominant inheritance, de novo mutation, fibroblast growth factor receptor 2 (FGFR2), syndactyly

## Abstract

**Rationale:**

Apert syndrome (AS) is an autosomal dominant inheritance pattern of the most severe craniosynostosis syndrome. AS is characterized by synostosis of cranial sutures and acrocephaly, including brachycephaly, midfacial hypoplasia, and syndactyly of the hands and feet. Patients with AS often present with craniosynostosis, severe syndactyly, and skin, skeletal, brain, and visceral abnormalities.

**Patient concerns:**

A pregnant Chinese woman presented with a fetus at 23 + 5 weeks of gestation with suspected AS in a prenatal ultrasound examination. Following ultrasound, the pregnancy underwent spontaneous abortion. Gene sequencing was performed on the back skin of the dead fetus.

**Diagnosis:**

The diagnosis of AS was confirmed on the basis of clinical manifestations of the fetus, and a de novo mutation in the fibroblast growth factor receptor 2 (*FGFR2*) gene was identified.

**Interventions:**

The couple finally chose to terminate the pregnancy based on the ultrasonic malformations and the risk of the parents having a neonate with AS in the future is small. However, any future pregnancy must be assessed by prenatal diagnosis.

**Outcomes:**

The dead fetus presented with bilateral skull deformation. Additionally, there were bilateral changes to the temporal bone caused by inwards movement leading to concave morphology, a “clover” sign, and syndactyly from the index finger/second toe to the little finger/little toe. AS was diagnosed by genetic testing, which showed a p.S137W (c.410C>G, chr10:123279677) mutation in the *FGFR2* gene.

**Lessons:**

Clinicians should be aware that there are a variety of ultrasound findings for AS. Therefore, genetic testing should be used when appropriate to confirm diagnosis of AS.

## 1 Introduction

Apert syndrome (AS) is a rare genetic disorder, which was first reported by Wilkie et al and characterized by craniosynostosis, severe syndactyly, and a variety of skin, skeletal, brain, and visceral abnormalities.^[1]^ AS exhibits an autosomal dominant inheritance pattern and the incidence of AS has been reported to be 1 in 65,000 individuals.^[2,3]^

The primary cause of AS is mutation in the fibroblast growth factor receptor 2 (*FGFR2*) gene. In fact, more than 98% of AS cases are caused by *de novo FGFR2* mutations, referred to as S252W and P253R.^[1,4]^ The mechanism underlying the exquisite genotype/phenotype correlation associated with AS mutations needs to be understood in terms of the biology of fibroblast growth factor/receptor signaling and the structural pathophysiology of *FGFR2* mutations.^[5]^ The *FGFR2* gene is located on chromosome 10q26. *FGFR2* encodes a transmembrane receptor with an extracellular region comprising 3 immunoglobulin-like domains (IgI, IgII, and IgIII), a hydrophobic transmembrane segment, and a cytoplasmic tyrosine-kinase1 domain. The immunoglobulin domains are encoded by exons 8, 9, and 10 of the *FGR2* gene in which 25 to 75 of patients with AS have mutations.^[6]^

In this study, we report the case of a Chinese fetus at 23 + 5 weeks of gestation with unclear prenatal ultrasound findings (acrocephalosyndactyly) and abnormal physical characteristics. The fetus was subsequently diagnosed with AS on the basis of DNA sequencing analysis of *FGFR*. We detected a novel mutation in the *FGR2* gene, p.S137W (c.410C>G chr10:123279677).

## 2 Case report

On 29 December 2016, a 32-year-old pregnant Chinese woman was admitted to the Prenatal Diagnosis Department of the First Hospital of Changchun, Jilin Province, northeastern China. She had previously undergone routine prenatal screening of her fetus (23 + 5 weeks of age). However, prenatal ultrasound findings were not clear. The woman had experienced a spontaneous abortion during the first trimester (8 weeks of gestation) of a previous pregnancy but without any known cause. She had not been exposed orally to harmful or hazardous substances during her pregnancies. During the second pregnancy, the pregnant woman had a febrile illness and received oral cold medication, although no specific records were available to confirm this. Following ultrasound, the second pregnancy underwent spontaneous abortion. The parents wanted to know the reason for the spontaneous abortion and brought the fetus to our department for analysis. There was no history of consanguineous marriage in the family and no family history of genetic disorders.

The study protocol was approved by the Ethics Committee of the First Hospital of Jilin University (No. 2016-365). Informed written consent was obtained from the parents for publication of this case report and accompanying images.

## 3 Materials and methods

Sequencing analysis was conducted on the dead fetus and the parents. First, the DNA was disrupted and a library was prepared. DNA of the targeted gene coding region and the adjacent cleavage region was then captured and enriched by a chip. Finally, detection of mutations was performed using a high-throughput sequencing platform.

## 4 Results

**Figure 1:**
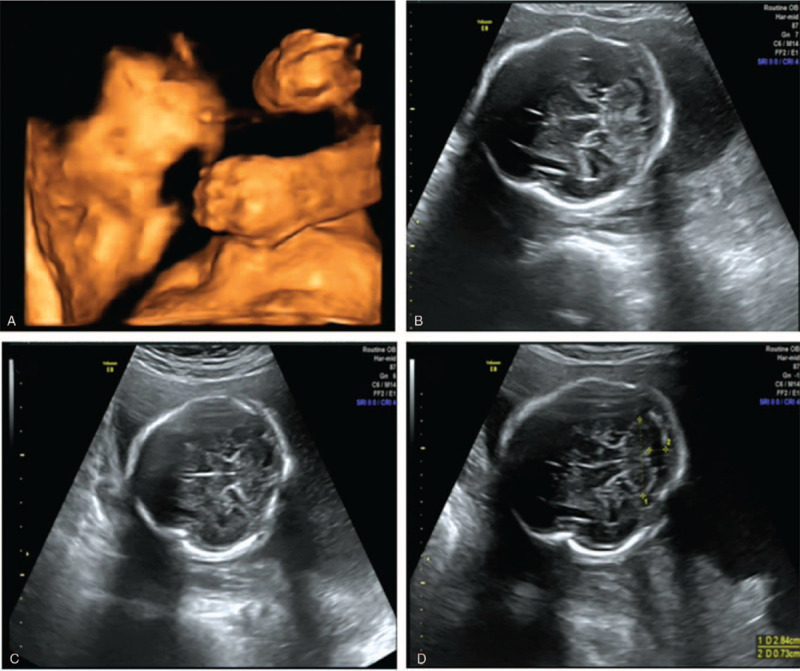
Prenatal ultrasound findings. (A) Syndactyly from the index finger to the little finger. (B–D) Physical characteristics of acrocephalia. The left ventricle was wider than normal.

During prenatal ultrasound, a number of anomalies were detected. These included a wide angle after the left ventricle (1.03 cm), bilateral skull deformation, bilateral temporal bone abnormalities showing inwards movement and a concave appearance, a “clover” sign, and syndactyly from the index finger/second toe to the little finger/little toe (Fig. 1).

**Figure 2:**
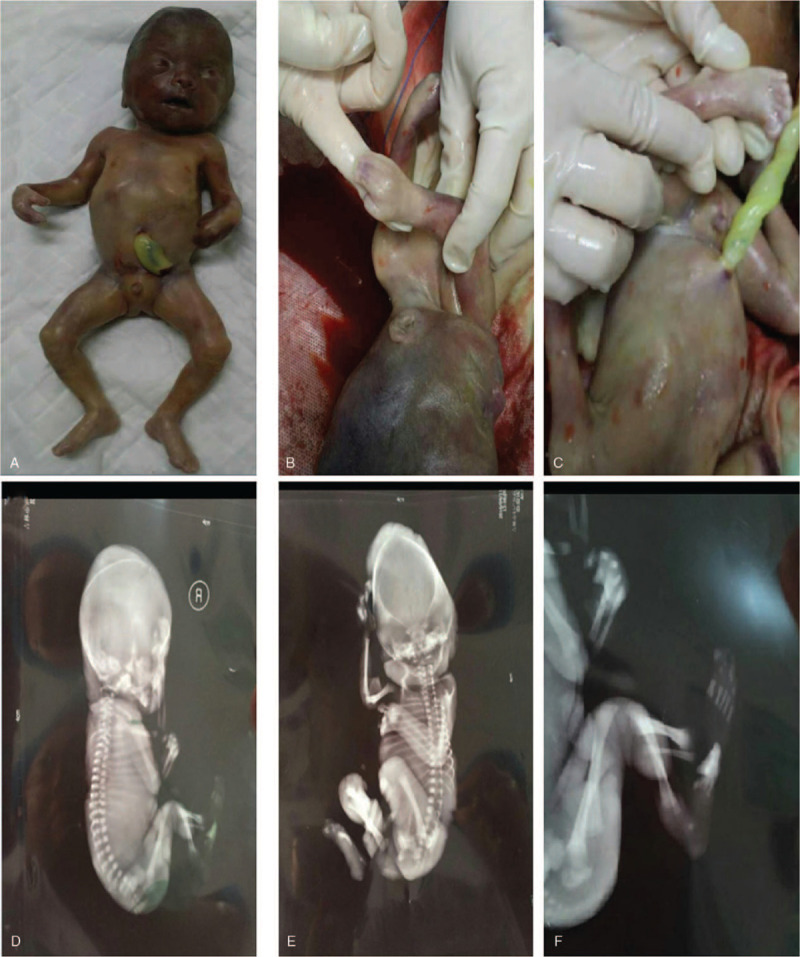
The dead fetus after induced labor. (A–C) Physical characteristics of acrocephalosyndactyly. (D–F) Presentation of acrocephalia as shown by X-ray. Craniosynostosis was not evident.

The physical characteristics of the dead fetus were consistent with the prenatal ultrasound findings (Fig. 2). The dead fetus presented with the physical characteristics of acrocephalosyndactyly and hypertelorism. X-ray results showed that there was no craniosynostosis. Because of the prenatal ultrasound findings and the clinical manifestations evident upon physical examination of the dead fetus, we initially suspected that the fetus may have had AS.

**Figure 3:**
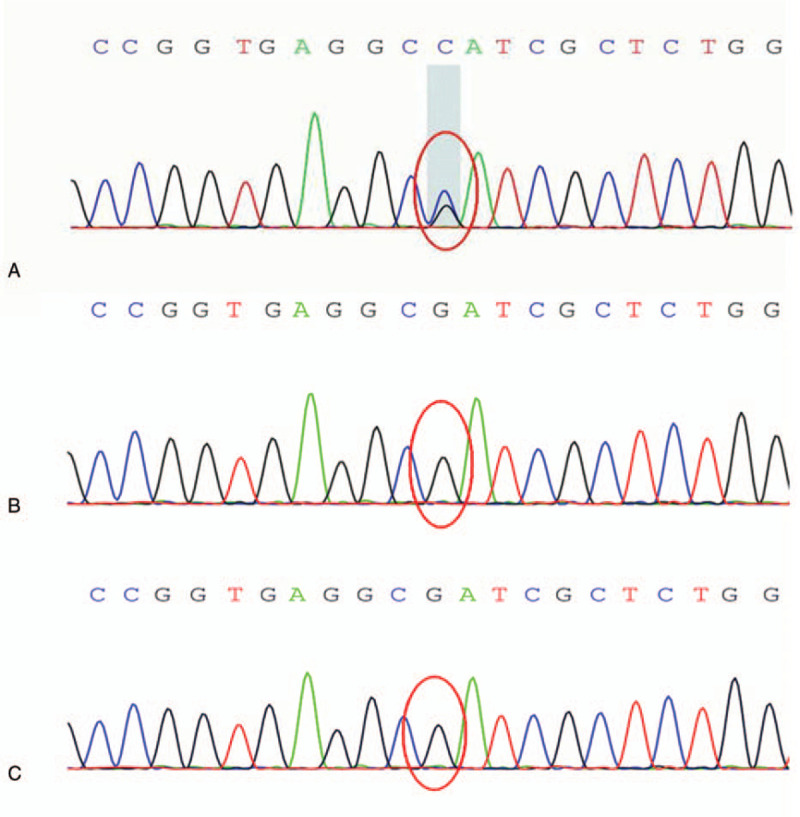
Sanger sequencing results for (A) the fetus, (B) the fetus's father, and (C) the fetus's mother. We identified the presence of a p.S137W (c.410C>G chr10:123279677) mutation in the *FGFR2* gene (red circle) in the fetus and the absence of a mutation at c.410C (red circles) in the parents.

We took a sample of skin from the back of the dead fetus for gene sequencing. We found that the fetus had a heterozygous p.S137W (c.410C>G, chr10:123279677) mutation in the *FGFR2* gene (Fig. 3A). However, further analysis showed that neither of the parents carried this mutation (Fig. 3B and C). The diagnosis of AS was confirmed on the basis of clinical manifestations of the fetus and identification of a de novo mutation in the *FGFR2* gene.

## 5 Discussion

We report here a new case of the rare genetic syndrome AS. AS is primarily characterized by cutaneous and osseous symmetric syndactyly in the hands and feet, with variable presentations in the bones, brain, skin, and other internal organs. AS is one of the most severe craniosynostosis syndromes.

During craniofacial development at different stages of embryonic formation, signaling pathways involving fibroblast growth factors, their receptors (FGFRs), and specifically, FGFR2, regulate the balance between proliferation and differentiation of progenitor osteogenic cells on the neural crest.^[7]^ Later in development, these pathways are also involved in formation of cartilage, skull bones, and the maxilla, as well as migration of the plates that give rise to the palate and lips.^[8]^

The S252W and P253R mutations were originally described by Wilkie et al^[1]^and are associated specifically with AS. These mutations present with more severe syndactyly in patients with the Pro253Arg mutation compared with the Ser252Trp mutation. However, the fetus described above showed no association of these mutations and syndactyly. Instead, the fetus had a novel mutation, which, to the best of our knowledge, has not been reported previously. Molecular analysis detected a p.S137W (c.410C>G chr10:123279677) mutation in the *FGFR2* gene. Because the parents of the fetus did not have this mutation, the fetus had a de novo heterozygous mutation, which led to AS.

Early surgery is currently advocated by many craniofacial centers to prevent complications arising from AS.^[9]^ Generally, the most important management protocol for patients with AS involves immediate craniotomy, hand and feet surgery, and long-term follow-up. However, in most developing countries, early intervention of AS is hampered by late diagnosis, a lack of facilities, and financial constraints.^[10]^

## 6 Conclusion

In conclusion, clinicians should be aware that there are a variety of ultrasound findings for AS. In the present study, the fetus showed abnormal prenatal ultrasound findings (acrocephalosyndactyly). However, craniosynostosis was not clearly indicative of AS. Therefore, genetic testing should be used when appropriate to confirm diagnosis of AS. Our data indicate that the case of fetal AS was caused by a new mutation. The risk of the parents having a neonate with AS in the future is small. However, any future pregnancy must be assessed by prenatal diagnosis.

## Author contributions

**Conceptualization:** Yanhong Liu.

**Data curation:** Qingyang Shi.

**Funding acquisition:** Ruizhi Liu.

**Investigation:** Qingyang Shi, Ruixue Wang.

**Methodology:** Xiaowei Yu.

**Software:** Jili Jing.

**Writing – original draft:** Rulin Dai.

**Writing – review & editing:** Qingyang Shi, Yanhong Liu.
